# Update on Nonsurgical Lung Volume Reduction Procedures

**DOI:** 10.1155/2016/6462352

**Published:** 2016-05-17

**Authors:** J. Alberto Neder, Denis E. O'Donnell

**Affiliations:** Division of Respiratory and Critical Care Medicine, Department of Medicine, Queen's University and Kingston General Hospital, Kingston, ON, Canada K7L 2V6

## Abstract

There has been a surge of interest in endoscopic lung volume reduction (ELVR) strategies for advanced COPD. Valve implants, coil implants, biological LVR (BioLVR), bronchial thermal vapour ablation, and airway stents are used to induce lung deflation with the ultimate goal of improving respiratory mechanics and chronic dyspnea. Patients presenting with severe air trapping (e.g., inspiratory capacity/total lung capacity (TLC) < 25%, residual volume > 225% predicted) and thoracic hyperinflation (TLC > 150% predicted) have the greatest potential to derive benefit from ELVR procedures. Pre-LVRS or ELVR assessment should ideally include cardiological evaluation, high resolution CT scan, ventilation and perfusion scintigraphy, full pulmonary function tests, and cardiopulmonary exercise testing. ELVR procedures are currently available in selected Canadian research centers as part of ethically approved clinical trials. If a decision is made to offer an ELVR procedure, one-way valves are the first option in the presence of complete lobar exclusion and no significant collateral ventilation. When the fissure is not complete, when collateral ventilation is evident in heterogeneous emphysema or when emphysema is homogeneous, coil implants or BioLVR (in that order) are the next logical alternatives.

## 1. Clinical Problem

The efficacy of pharmacological approaches in promoting lung deflation in COPD is limited when the main mechanism of lung hyperinflation is no longer bronchial constriction and airway narrowing but the anatomical consequences of extensive alveolar destruction. Ever since the encouraging results of the landmark National Emphysema Treatment Trial (NETT), there has been a surge of interest in novel nonsurgical lung volume reduction (LVR) strategies for advanced COPD. Endoscopic procedures (ELVR) (Table 1) [1–4], in particular, have gained momentum due to the excess morbidity and mortality found in some specific NETT subgroups. By promoting lung deflation, these procedures are aimed at improving respiratory mechanics with the ultimate goal of ameliorating the distressing symptom of chronic dyspnea. Unfortunately, there remains a lack of evidence-based recommendations to assist the selection of patients who are most likely to benefit from various current interventions. In order to help the clinician decide on the best option for individual patients, this focused review will critically appraise the current evidence on the topic. We recovered pertinent publications in English that were retrieved from PubMed*™* up to May 2015, with particular consideration of randomized controlled trials (RCTs) and meta-analyses.

## 2. Physiological Rationale for Lung Deflation in COPD

Severe lung hyperinflation places the inspiratory muscles, especially the diaphragm, at a significant mechanical disadvantage by shortening its fibers and compromising its force generating capacity. The increase in dyspnea intensity at any given ventilation during exercise in advanced COPD ultimately reflects the inability of the compromised respiratory system to respond appropriately to increasing respiratory neural drive, that is, neuromechanical dissociation [5]. It follows that reduction in lung hyperinflation following endoscopic LVR should help reduce respiratory discomfort.

While surgical LVR (SLVR) excises lung areas of predominant high ventilation/perfusion ratios, endoscopic LVR (ELVR) may decrease or, ideally, obliterate ventilation to those areas. Thus, physiological dead space is expected to decrease in response to effective LVR and, with it, respiratory neural drive and ventilatory requirements for a given external power output. Improvements in cardiopulmonary interactions may also occur due to enhancement of venous return and lower right ventricle afterload with benefits for left ventricular filling, When these mechanical and cardiocirculatory improvements are coupled with reduced respiratory neural drive (due to improved pulmonary gas exchange), the net effect is reduced neuromechanical dissociation of the respiratory system and improved activity-related dyspnea.

### 2.1. Endoscopic One-Way Valve Implantation

The one-way valves are intended to work by preventing inspired air from entering target airways whilst allowing exit of trapped air from distal airways (Table 1). The umbrella-like “intrabronchial valve” (IBV) is deployed bilaterally to the upper lobes to redistribute ventilation to less emphysematous areas. The mouth-fish appearing “endobronchial valve” (EBV) is deployed unilaterally to induce total lobar atelectasis. There is growing evidence that EBV is more effective than IBV [1]. Regardless of the valve that is used, best results are obtained in heterogeneous emphysema when lobar ventilation can be isolated; that is, there is little collateral ventilation. Heterogeneity can be defined, for instance, as the difference in the quantitative emphysema score between the targeted lobe and the adjacent ipsilateral nontargeted lobe. A key anatomical feature associated with lung deflation, therefore, is a “complete” fissure as suggested by the absence of a parenchymal bridge connecting the lobes for >10% of the fissure [2]. When both complete fissure and lobar occlusion are present, substantial increments in FEV_1_ (up to 26%, on average) have been reported in 6 months and sustained at 12 months. It should be noted that only ~20% of patients met these strict criteria. Assessment of fissure integrity also requires radiological expertise but specific software packages are available. There is recent evidence, however, that CT scans overestimate completeness of the right minor fissure and underestimate completeness of the right major fissure [25]. Alternatively, or in addition, a dedicated endobronchial balloon and flow-transducer system can be used to assess collateral ventilation.

The most recent meta-analyses showed that one-way valves were associated with minor, but significant, increases in mean FEV_1_ (~7%) compared to standard medical care in patients with severe to very severe COPD (Table 2) [3, 4]. Statistically significant changes were also seen in chronic dyspnea; however, improvements were modest and only marginally greater than the minimally important difference (MID). Increases in peak work rate during an incremental cycle test were also significant (~5 W) but also lower than the suggested MID (10 W). Six-minute walking distance failed to significantly increase. The rate of adverse events tended to be greater with EBV but this was mostly related to nonmassive hemoptysis and, less commonly, pneumothorax and pneumonia [3, 4]. Unfortunately, effective postprocedure lobar atelectasis which might be associated with better functional results was also associated with a greater incidence of pneumothorax.

### 2.2. Coil Implants

With this method, a deployed coil conforms to a predetermined shape (“memory-shape” coil). By bending in the airway and causing compression of adjacent lung tissue, it induces local LVR (intrabronchial coil). Alternatively, multiple endobronchial coils may be implanted throughout a lobe achieving deflation through increased radial tension across the airway network which might also open small airways by increased tethering effects. A potential advantage is that the implants do not depend on (the absence of) collateral ventilation and therefore could be useful for patients with relatively homogeneous emphysema (Table 1). Conversely, patients with large bullae are unlikely to benefit from this technique since the proposed mechanism is shortening of the airways. Effectiveness and a good safety profile have been reported in small cohorts of patients with heterogeneous emphysema. A single, relatively small RCT involving patients with heterogeneous and homogeneous disease found a significant improvement in quality of life which was maintained up to one year following procedure. This was accompanied by improvements in FEV_1_ and decrements in pulmonary gas trapping (but not total lung capacity (TLC)) [16]. The most frequently reported adverse events were pneumonia and COPD exacerbation, both <10% (Table 2).

### 2.3. Biological LVR (BioLVR)

BioLVR aims to promote LVR through intra-airway polymerization of fibrinogen suspension and thrombin solution with the purpose of inducing a localized inflammatory reaction (Table 1) [1]. The ensuing irreversible atelectasis and tissue remodeling are expected to induce lung contraction and volume reduction in 1-2 months. An alternative Japanese approach using autologous blood and thrombin has been used in a small number of selected patients with very severe COPD [26]. The biological sealants can also work regardless of the integrity of the interlobar fissure [1]. Several observational or pilot studies found positive effects on lung hyperinflation, exercise tolerance, and quality of life with an acceptable safety profile (Table 2). The first RCT with BioLVR confirmed these preliminary findings regarding efficacy [27]. Unfortunately, the study was prematurely terminated for business-related reasons after only 95 of the planned 300 were randomized. Of note, despite only 2 deaths, the number of serious adverse events was markedly greater in the treatment versus control groups.

### 2.4. Bronchial Thermal Vapour Ablation (BTVA) Therapy

BTVA uses heated water (steam) to produce thermal injury of the target tissue, usually a segmental airway. Similar to BioLVR, the treatment aims to induce lung volume reduction regardless of the presence of collateral ventilation (Table 1). As expected, patients with higher inflammatory responses can achieve better clinical outcomes. More experience has been gained with patients showing heterogeneous upper lobe-predominant emphysema who do not present with a bulla of more than a third of the lobar volume. The largest multicenter trial to date reported improved lung function, exercise tolerance, and quality of life (Table 2) [21]. The magnitude of these benefits, however, lessened at 6 months, suggesting progression of COPD or compensatory hyperinflation. Serious adverse events were observed: COPD exacerbation, pneumonia, and respiratory tract infection were the most common complications. A multicenter, randomized trial evaluating safety and efficacy following segmental, bilateral BTVA in patients with severe emphysema is underway in Europe and Australia and results are expected in 2015/2016.

### 2.5. Airway Bypass Stents

Airway bypass stents have been used to create and maintain passages between the bronchi and emphysematous lobes. Efficacy of the technique, therefore, depends strongly on the lack of collateral ventilation (Table 1). Despite promising initial results, the largest trial to date (*n* = 208) failed to show significant improvement in the main functional outcomes at 1, 3, 6, and 12 months (Table 2) [23, 24]. Moreover, the stents were lost in most cases possibly due to chronic cough and expectoration. There was also significant granulation and occlusion in remaining stents. A recent meta-analysis confirmed that, among the available endoscopic approaches, the stents had the least impressive performance to date [3].

## 3. Recommendations

**Figure 1:**
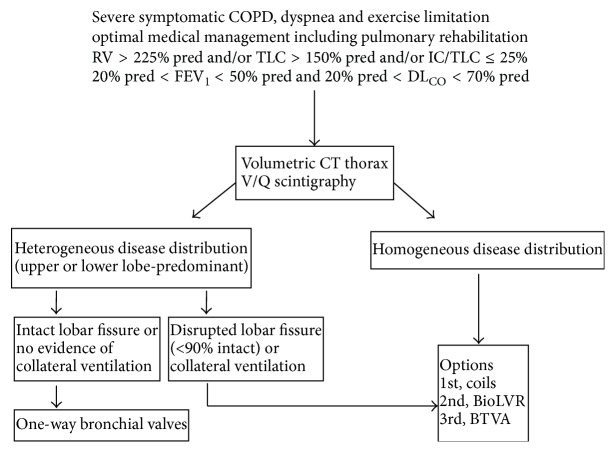
Algorithm for endoscopic LVR evaluation and selection of procedure. BioLVR: biological lung volume reduction; BTVA: bronchial thermal vapour ablation; CT: computed tomography; IC: inspiratory capacity; pred: predicted; RV: residual volume; TLC: total lung capacity; V/Q: ventilation/perfusion.

(i) Patients presenting with severe air trapping (e.g., inspiratory capacity (IC)/TLC < 25%, residual volume > 225% predicted) and thoracic hyperinflation (TLC > 150% predicted) have the greatest potential to derive benefit from ELVR procedures (Figure 1). Very severe functional impairment (FEV1 and/or DL_CO_ ≤ 20% predicted) is an established contraindication for ELVR.
(ii) Pre-LVRS or ELVR assessment should ideally include cardiological evaluation, high resolution CT scan, ventilation and perfusion scintigraphy, full pulmonary function tests, cardiopulmonary exercise testing, and measurements of quality of life and dyspnea (Figure 1). Careful computer-based assessment of fissure integrity on chest CT and endobronchial balloon-occluding systems to assess distal flow are recommended before ELVR.
(iii) If a decision is made to offer an ELVR procedure, one-way valves are the first option in the presence of complete lobar exclusion and no significant collateral ventilation (Figure 1). It should be expected, however, that only 1 in 5 eligible patients will meet these anatomic features. When the fissure is not complete, when collateral ventilation is evident in heterogeneous emphysema or when emphysema is homogeneous, coil implants or BioLVR (in that order) are the next logical alternatives. These nonreversible techniques (BioLVR and BTVA) appear to be less desirable owing to greater risk of persistent harm to already-frail patients. Nevertheless, benefits may accrue in highly selected patients in specialized centers. Currently, there appears to be insufficient evidence to support the use of airway bypass stents in the management of advanced emphysema.
(iv) No ELVR procedures have been approved by Health Canada. To date (June 2015), they are available except in research centers as part of clinical trials.

## Figures and Tables

**Table 1:** Overview of the currently available procedures for lung volume reduction (LVR) in advanced emphysema.

Technique	Dependence on collateral ventilation	Reversibility	Mechanisms of action	Principal complications
Valve implantation	Yes	Fully reversible	Prevention of inspired air from entering target airways whilst allowing exit of trapped air	Pneumothorax, hemoptysis

Coil implantation	No	Partially reversible (within 4 weeks)	Torquing of the bronchi (intrabronchial) Increased radial tension of adjacent airway network (endobronchial)	Hemoptysis, COPD exacerbations

Bronchoscopic thermal vapour ablation	No	Irreversible	Inflammatory reaction	Local and systemic inflammatory reaction

Airway stent	Yes	Partially reversible	Bypassing airway	Stent loss, stent obliteration

**Table 2:** Characteristics and outcomes of the larger published studies on endoscopic LVR for advanced emphysema (references [6–24]).

	Author, year	Study design	Patient population	Time point	ΔFEV_1_	Δ6-MWD	ΔSGRQ (units)
Valves	Wan et al. 2006 [6]	Prospective multicenter registry	*n* = 98	90 days	11 ± 3%	37 ± 90 m	—
	Sciurba et al. 2010 [7]	RCT	Treatment group (*n* = 214)	6 months	4%	9 m	−3
			Complete fissure (*n* = 68)		16%	8 m	—
			High heterogeneity (*n* = 90)		11%	12 m	—
	Sterman et al. 2010 [8]	Multicenter prospective cohort study	*n* = 91	12 months	−2 ± 12%	14 ± 104 m	−8.2 ± 16
	Herth et al. 2012 [9]	RCT	Treatment group (*n* = 111)	6 months	7 ± 20%	15 ± 91 m	−5 ± 14
			Complete fissure, lobar occlusion (*n* = 20)		26 ± 24%	22 ± 38%	−10 ± 15
	Eberhardt et al. 2012 [10]	Prospective, randomized, noncontrolled	Complete unilateral occlusion (*n* = 11)	3 months	21 ± 11%	49 ± 53 m	−12 ± 11
			Partial bilateral occlusion (*n* = 11)		−3 ± 15%	−52 ± 81 m	2 ± 9
	Ninane et al. 2012 [11]	RCT	Partial occlusion (*n* = 37)	3 months	−90 mL	7 m	−4
	Herth et al. 2013 [12]	Prospective, noncontrolled	CV negative (*n* = 51)	1 month	16 ± 22%	24 ± 57 m	−10 ± 13
			CV positive (*n* = 29)		1 ± 15%	10 ± 57 m	−5 ± 15
	Wood et al. 2014 [13]	RCT	Treatment group (*n* = 142)	6 months	−2 ± 5% pred	−24 ± 69 m	2 ± 16

Coils	Slebos et al. 2012 [14]	Prospective, noncontrolled	*n* = 16	6 months	15 ± 17%	84 ± 73 m	−15 ± 12
	Shah et al. 2013 [15]	RCT	Treatment group (*n* = 23)	3 months	14%	52 m	−8
	Zoumot et al. 2015 [16]	RCT	Treatment group (*n* = 35)	12 months	9 ± 22%	34 ± 52 m	−6 ± 14

BioLVR	Criner et al. 2009 [17]	Open-label, multicenter, non-RCT	Low-dose hydrogel (*n* = 28)	6 months	6.7 ± 12.9%	25.5 ± 53.2 m	−6.9 ± 8.8
			High-dose hydrogel (*n* = 22)		15.6 ± 16.8%	9.9 ± 51.2 m	−9.7 ± 18.8
	Herth et al. 2010 [18]	Open-label, multicenter, non-RCT	*n* = 21	3 months	3.3 ± 3.2%	10.8 ± 8.8%	−7.8 ± 3.7
	Magnussen et al. 2012 [19]	Retrospective analysis from multicenter non-RCTs	*n* = 28	12 weeks	19.1 ± 21.5% (0.18 ± 0.22 L)	30.9 ± 50.2 m	−11.6 ± 12.4
	Kramer et al. 2012 [20]	Multicenter open-label non-RCT	*n* = 18	12 months	25.0 ± 33.4%	8.6 ± 65.2 m	−7.0 ± 15.8

BTVA	Snell et al. 2012 [21]	Prospective, noncontrolled	*n* = 44	6 months	17%	47 m	−14
	Herth et al. 2012 [22]	Two multicenter single-arm prospective studies	*n* = 37	12 months	86 ± 174 mL	18.5 ± 63.7 m	−11 ± 14

Stents	Cardoso et al. 2007 [23]	Multicenter non-RCT	*n* = 36	6 months	0.6%	−12 m	−1.8
	Shah et al. 2011 [24]	Multicenter RCT	*n* = 208	12 months	−20 ± 200 mL	−21 m	−1
					−0.15 ± 7%		

Values for changes (Δ) are means ± SD.

6-MWD: 6 min walking distance; BTVA: bronchial thermal vapour ablation; FEV_1_: forced expiratory volume in one second; RCT: randomized controlled trial; SGRQ: St. George's Respiratory Questionnaire.
